# Two *Streptococcus pyogenes emm* types and several anaerobic bacterial species are associated with idiopathic cutaneous ulcers in children after community-based mass treatment with azithromycin

**DOI:** 10.1371/journal.pntd.0011009

**Published:** 2022-12-19

**Authors:** Brad Griesenauer, Yue Xing, Katherine R. Fortney, Xiang Gao, Camila González-Beiras, David E. Nelson, Jie Ren, Oriol Mitjà, Qunfeng Dong, Stanley M. Spinola

**Affiliations:** 1 Department of Microbiology and Immunology, Indiana University School of Medicine, Indianapolis, Indiana United States of America; 2 Department of Medicine, Stritch School of Medicine, Loyola University Chicago, Maywood, Illinois United States of America; 3 Carretera de Canyet, Hospital Universitari Germans Trias i Pujol, Barcelona, Spain; 4 Department of Biostatistics and Health Data Science, Indiana University School of Medicine, Indianapolis, Indiana United States of America; 5 Department of Medicine, Indiana University School of Medicine, Indianapolis, Indiana United States of America; 6 Department of Pathology and Laboratory Medicine, Indiana University School of Medicine, Indianapolis, Indiana United States of America; International Atomic Energy Agency, AUSTRIA

## Abstract

**Background:**

In yaws-endemic areas, two-thirds of exudative cutaneous ulcers (CU) are associated with *Treponema pallidum* subsp. *pertenue* (TP) and *Haemophilus ducreyi* (HD); one-third are classified as idiopathic ulcers (IU). A yaws eradication campaign on Lihir Island in Papua New Guinea utilizing mass drug administration (MDA) of azithromycin initially reduced but failed to eradicate yaws; IU rates remained constant throughout the study. Using 16S rRNA gene sequencing, we previously determined that *Streptococcus pyogenes* was associated with some cases of IU. Here, we applied shotgun metagenomics to the same samples we analyzed previously by 16S rRNA sequencing to verify this result, identify additional IU-associated microorganisms, and determine why *S*. *pyogenes*-associated IU might have persisted after MDA of azithromycin.

**Methodology/Principal findings:**

We sequenced DNA extracted from 244 CU specimens separated into four groups based upon microorganism-specific PCR results (HD+, TP+, TP+HD+, and TP-HD- or IU). *S*. *pyogenes* was enriched in IU (24.71% relative abundance [RA]) specimens compared to other ulcer sub-groups, confirming our prior results. We bioinformatically identified the *emm* (M protein gene) types found in the *S*. *pyogenes* IU specimens and found matches to *emm156 and emm166*. Only ~39% of IU specimens contained detectable *S*. *pyogenes*, suggesting that additional organisms could be associated with IU. In the sub-set of *S*. *pyogenes*-negative IU specimens, *Criibacterium bergeronii*, a member of the *Peptostreptococcaceae*, and *Fusobacterium necrophorum* (7.07% versus 0.00% RA and 2.18% versus 0.00% RA, respectively), were enriched compared to the *S*. *pyogenes*-positive sub-set. Although a broad range of viruses were detected in the CU specimens, none were specifically associated with IU.

**Conclusions/Significance:**

Our observations confirm the association of *S*. *pyogenes* with IU in yaws-endemic areas, and suggest that additional anaerobic bacteria, but not other microorganisms, may be associated with this syndrome. Our results should aid in the design of diagnostic tests and selective therapies for CU.

## Author summary

The World Health Organization classifies yaws as a neglected tropical disease. It has been estimated that 100,000 children in 14 yaws-endemic countries develop cutaneous ulcers annually. The overall burden of infectious skin ulcers has not been determined and is likely to be much higher. Two-thirds of these ulcers are associated with *Treponema pallidum* subspecies *pertenue* and/or *Haemophilus ducreyi*, while the cause(s) of the remaining one-third are unknown. An ulcer eradication campaign on Lihir Island, Papua New Guinea, utilized mass drug administration of azithromycin and failed to fully eradicate ulcers in part due to the persistence of ulcers of unknown cause. By 16S rRNA gene sequencing, which only detects a subset of bacteria, we previously showed *S*. *pyogenes* was associated with skin ulcers of unknown origin. Here we applied direct DNA sequencing to identify other microorganisms present in these specimens. Confirming and extending our previous data, we found that *Streptococcus pyogenes* and several anaerobic bacteria were enriched in ulcers of unknown cause. We did not find enrichment of any viral or fungal microbes. Testing for *S*. *pyogenes* and selected anaerobes will help strengthen eradication efforts by improving diagnostic testing and treatment of this painful and debilitating syndrome.

## Introduction

Up to five to fifteen percent of children residing in tropical countries of the South Pacific islands and equatorial Africa have infectious cutaneous ulcers (CU), located primarily on the lower legs [1,2] Untreated, CU can cause chronic disfigurement and disability. The sole etiology of CU was once thought be *Treponema pallidum* subspecies *pertenue* (TP), the causative agent of yaws. More recently, *Haemophilus ducreyi* (HD), the causative agent of chancroid, has been also identified as an etiological agent of CU [2,3]. However, no TP or HD DNA can be detected by PCR in ~30% of CU cases, and the etiology of these idiopathic ulcers (IU) has yet to be entirely defined [4,5].

The World Health Organization (WHO) considers yaws to be a neglected tropical disease that should be eradicated [6]. As oral azithromycin is as effective as injectable benzathine penicillin in the treatment of yaws [7], the WHO initially launched a yaws eradication campaign based on the Morges Strategy, which includes diagnostic sampling of CU, mass drug administration (MDA) of a single dose of oral azithromycin to the entire community, and case finding and treatment of subsequent CU cases and their household contacts with azithromycin every 6 months [8]. This strategy was trialed on Lihir Island, Papua New Guinea. Twelve months post-MDA, CU prevalence decreased from 5.1% to 0.9% and TP prevalence decreased from 1.8% to 0.1% [9,10]. Although this decrease in TP was sustained through 24 months, by 42 months TP prevalence significantly increased to 0.4% and the overall prevalence of CU was still 0.8%. Since the overall prevalence of CU was still at 0.8% after 42 months, the trial was halted due to the failure to achieve the primary endpoint, eradication of yaws [10]. Another yaws eradication community-based trial in the Namatanai District of the New Ireland Province of Papua New Guinea used three rounds of MDA of azithromycin given every six months; however, this trial also failed to eradicate yaws [11].

Factors that could have contributed to these failures of prior attempts to eliminate yaws using MDA were the re-introduction of yaws in persons who were absent at the time of MDA, the emergence of azithromycin resistance in TP, environmental reservoirs of HD, and the consistent prevalence of IU throughout both trials [10,11]. The last observation suggests that azithromycin-resistant pathogen(s) could cause IU. Identification of these pathogens would help in designing studies for future CU eradication efforts.

Two prior attempts to identify IU-associated microorganisms in specimens from the Lihir Island cohort have been reported. The first study analyzed residual DNAs from CU specimens obtained 6 and 18 months post-MDA using a shotgun metagenomic sequencing approach. Sequences from *Corynebacterium diphtheriae*, *Arcanobacterium haemolyticum*, and *Streptococcus dysgalactiae* were enriched in the IU specimens compared to the TP and HD specimens [4]; however, this study was limited by the use of residual samples that were not collected specifically for microbiome analysis, sequencing depth, and the annotation methods used. The second study analyzed CU specimens prospectively collected specifically for PCR testing and microbiome analysis 36, 42, and 48 months post-MDA. Using 16S rRNA gene sequencing, a single amplicon sequence variant (ASV) of *Streptococcus pyogenes* was enriched in IU and in non-healing ulcers compared to TP, HD, and TP/HD ulcers and ulcers which showed signs of healing, respectively [5]. However, 16S rRNA gene sequencing can skew bacterial abundance, can only detect bacteria that retain sufficient homology to the degenerate PCR primers employed, and cannot detect other types of microorganisms [12]. As well, *S*. *pyogenes* was not detected in over half of the IU specimens [5]. Thus, although both studies suggested that bacterial pathogens were associated with IU, they were limited by their inability to detect a broad range of microorganisms and failed to identify IU associated microorganisms in many IU specimens.

Clinical management of CU and future eradication efforts would be greatly improved by identifying all causes of CU in endemic communities. Here, we applied shotgun metagenomic sequencing to detect a broader range of microorganisms in the same CU samples prospectively collected 36, 42, and 48 months post-MDA from the Lihir Island cohort that we had analyzed by 16S rRNA gene sequencing previously [5]. Our goals were to confirm that *S*. *pyogenes* is associated with IU and to identify other possible bacterial and non-bacterial agents of IU. If *S*. *pyogenes* was confirmed to be associated with IU, another goal was to determine the *emm* type of the circulating strains.

## Materials and methods

### Ethics statement

The study protocol was approved by the National Medical Research Advisory Committee of the Papua New Guinea Ministry of Health (MRAC number 12.36). Written informed consent was obtained from all participants, or their parents or guardians, and verbal agreement from the children was obtained before enrollment.

### Participants

The participants were part of a prospective cohort study of a yaws eradication campaign on Lihir Island, Papua New Guinea, as described previously [5]. Participants with papillomatous skin lesions or atraumatic skin ulcers that measured > 1 cm were eligible for the study. S1 Table summarizes demographics of the participants.

### Specimen collection

As part of the yaws eradication campaign, 83% of Lihir Island inhabitants were given a single dose of oral azithromycin (30 mg/kg; max 2g) and were re-examined every six months for up to 48 months. Participants who subsequently developed CU had their ulcers swabbed and they and their household contacts were treated with azithromycin. Although the trial was halted at 42 months, specimens were collected from a sub-set of the participants who had CU at 48 months post-MDA to monitor for azithromycin resistance in TP.

Using techniques to minimize contamination, we prospectively obtained 279 CU specimens at 36, 42, and 48 months (May 2016, N = 106; November 2016, N = 107; and May 2017, N = 66, respectively) post-MDA. Specimens were collected by vigorously rubbing the base of the ulcer with a sterile, dry, Dacron-tipped swab, which was inserted into Eppendorf containing 0.5 mL lysis buffer (10mM Tris-HCl, 0.1M EDTA, 0.5% SDS) [5]. The specimens were shipped from the study site to Indiana University Purdue University Indianapolis, where an aliquot of the lysate was removed and stored at -80°C until DNA was extracted for metagenomic sequencing. The remaining sample was shipped to the University of Washington (Seattle) where real-time PCR for detection of HD and/or TP DNA was performed.

### PCR and real-time PCR

To identify HD or TP DNA in ulcer specimens, real-time PCR was initially performed on the extracted DNA using a multiplex TaqMan assay, utilizing TaqMan MGB probes (Thermo Scientific, Carlsbad, CA, USA) with 4–5 replicates, as described in detail elsewhere [13,14]. A specimen was considered positive for TP and/or HD if Cycle threshold (Ct) values were below 40 for at least 1 of the replicates amplified. DNAs from specimens that were initially negative for HD and/or TP were precipitated and concentrated, and PCR was repeated on the concentrated specimens with a positive result for HD and/or TP being called if Ct values were below 40 for at least 1 of the replicates amplified. As a DNA integrity control for specimens with no detectable HD or TP, human β-globulin DNA was amplified, as previously described [5]. No template and DNA extraction negative controls were included with each PCR run, and were never positive, over several hundred specimens.

### Shotgun metagenomic library preparation and sequencing

We extracted genomic DNA (gDNA) from the unconcentrated specimen lysate using the DNeasy Blood and Tissue Kit (Qiagen) with pre-treatment for Gram-positive bacteria, according to manufacturer’s protocol, except we used 40 mg/mL lysozyme to increase yield from Gram-positive organisms. (Research Products International). Positive controls (N = 4) consisted of a mock community skin control (BEI Resources HM-782D). Negative controls consisted of DNA isolation buffers, aliquots of lysis buffer used in the field, lysozyme, and water used during gDNA extraction. DNA concentrations were quantified on a Qubit 4 Fluorometer (Thermo Fisher Scientific) using the Quant-iT dsDNA Assay Kit, High Sensitivity (Invitrogen). Extracted DNA from the specimens were stored at 4°C until it was used to construct metagenomic sequencing libraries.

Shotgun metagenomic gDNA libraries were constructed using the Nextera XT DNA Library Preparation Kit (Illumina, Inc.), according to manufacturer’s protocol, from 1 ng gDNA. Dedicated lots of reagents were used in library preparation to minimize batch effects. Four pools of sequencing libraries were generated, with each pool containing a library constructed from skin mock community gDNA (BEI Resources catalog no. HM-782D). Pools were sequenced on an Illumina Novaseq 6000 platform using a S4 flow cell with 2 x 150 bp, paired end, at the Indiana University Medical Genomics Core, Indianapolis, Indiana. In all pools, the mock community controls yielded the expected bacterial sequences. Raw sequences, raw counts, and metadata were deposited in the Sequence Read Archive with the accession number PRJNA906500.

### Sequence processing

Raw sequence reads were annotated to taxonomic counts using MetaPhlAn3 [15]. Kraken2 [16] was used to count human sequences in the raw reads [17]. MetaPhlAn3 taxonomic counts were transformed by additive log transformation (ALR, log of micro-organism counts plus 0.1 divided by number of human reads of that subject).

### Sequencing analyses and statistics

Samples were classified and sorted by their PCR-determined etiology and were further grouped on Euclidean distances of ALR transformed microbial taxonomic counts, using the partitioning around medoids clustering algorithm (“pam” in R package “cluster”). The optimal number of clusters were discovered by a Calinski-Harabasz (CH) index based on ALR transformed dataset [18]. Heatmaps were generated using R packages (cluster, clusterSim, circlize, ComplexHeatmap, vegan, phyloseq and taxize) [19–25] and custom R scripts based on ALR transformed data and optimal number of clusters. Relative abundance was used for display purpose on heatmaps. Principal component analysis (PCA) was performed on ALR transformed data by R [26]. Permutational multivariate analyses of variance (PERMANOVA) were performed on the Euclidean distance matrix, derived from the ALR transformed taxa, to identify differences in microbe community composition between etiologies. Differential abundance between etiology groups, and between *S*. *pyogenes* positive and negative subjects in the IU group, were performed by Wilcoxon signed-rank test based on ALR transformed overall and stringent datasets. Alpha diversity of the groups were calculated using R package phyloseq.

### *emm* typing of *S*. *pyogenes* in IU specimens

Cataloged *emm* gene sequences were obtained from the Center for Disease Control and Prevention website (https://www2.cdc.gov/vaccines/biotech/strepblast.asp). BLAST was conducted for the *emm* genes against the raw reads of the 18 IU specimens containing *S*. *pyogenes*. Because few perfect matches (100% identity, no mismatch, no gap open) were found, a reference-based approach was conducted. The single ASV of *S*. *pyogenes* that was detected in our previous 16S rRNA gene study was used to select *S*. *pyogenes* reference genomes. BLAST was conducted for this ASV against all *S*. *pyogenes* assemblies in NCBI, and the perfect matches (100% identity with max scores) were collected. From them, sequences with perfect *emm* gene matches (length > 100 bp, 95% query coverage identity, no mismatches or gaps, 100% query coverage) were filtered out. The *emm* genes used in this BLAST were the perfect matched *emm* genes (length > 100 bp, 100% query coverage, no mismatches or gaps) collected when they were searched against human-removed raw reads of all subjects. The 7 strains filtered out were finally used as reference. The raw reads from the 18 IU specimens containing *S*. *pyogenes* were merged and reads that aligned to the seven reference *S*. *pyogenes* genomes were extracted by BWA [27]. Contigs, scaffolds and pseudochromosomes were then assembled from these data by SpaDES [28], BBMap [29], SSPACE [30], ragtag [31] and MeDuSa [32]. *emm* gene sequences were compared to these assemblies using BLAST, and perfect matches were kept.

## Results

### Specimens

Specimens collected prospectively during the yaws eradication campaign on Lihir island are described in detail in our previous study [5]. A specimen was defined as a single swab, placed in a lysis buffer, of one ulcer. For this study, we extracted DNA from specimens obtained from 279 unique participants collected between 36–48 months post-MDA of azithromycin (S1 Table).

### Classification of CU specimens

We initially classified CU specimens into TP, HD, both (TP/HD) DNAs, or neither DNA (IU) groups based upon the results of real-time multiplex PCR with pathogen-specific primers. Human β-globulin was used as a marker for DNA integrity as the specimens primarily contain host DNA [9,10]. Of the 279 CU specimens, 275 contained human β-globulin DNA; 244 of those contained sufficient genomic DNA for shotgun metagenomic sequencing. Of the 244 CU specimens, 79 were classified as HD+, 83 as TP+, 35 as TP+HD+, and 47 as IU by PCR.

MetaPhlAn3 was used to annotate the metagenomic sequences from the CU specimens to specific microorganisms. Similar to our prior findings using 16S rRNA gene sequencing data [5], the relative abundance (RA) of shotgun metagenomic HD and TP sequences was higher in non-IU specimens (11.60%-22.43%). Similar to our prior study [5], discrepancies were observed in the results of the PCR and shotgun sequencing; some specimens that were pathogen positive in one assay were negative in the other assay and vice versa (S2 Table). To account for this and to better compare the shotgun sequencing data to our previous 16S rRNA study, we applied the stringent classification system developed in our previous study [5]. Specimens that had >0.1% RA and >100 reads of either HD, TP, or both by 16S rRNA gene sequencing and in which ≥75% of wells yielded a positive PCR result for HD, TP, or both were classified as HD positive (HD+), TP+, or TP+HD+. Specimens that had <0.1% RA and <100 reads and were PCR negative for both pathogens were classified as IU. Specimens that did not meet all these criteria were excluded from the stringent dataset. The stringent dataset contained 139 specimens consisting of 35 HD+, 49 TP+, 9 TP+HD+, and 46 IU samples (S3 Table); shotgun sequencing correctly identified the presence or absence of HD and/or TP sequences in these samples. Further analyses were performed on both the stringent and overall (etiology defined by detection of pathogen(s) via PCR) datasets. Results from both datasets were similar; therefore, figures from the stringent dataset are in the main text and figures from the overall dataset are in the supplemental material. Relative abundances of all microbial taxa identified in the stringent and overall datasets are in the files S1 Data and S2 Data, respectively.

### Ulcer microbiomes

**Fig 1:**
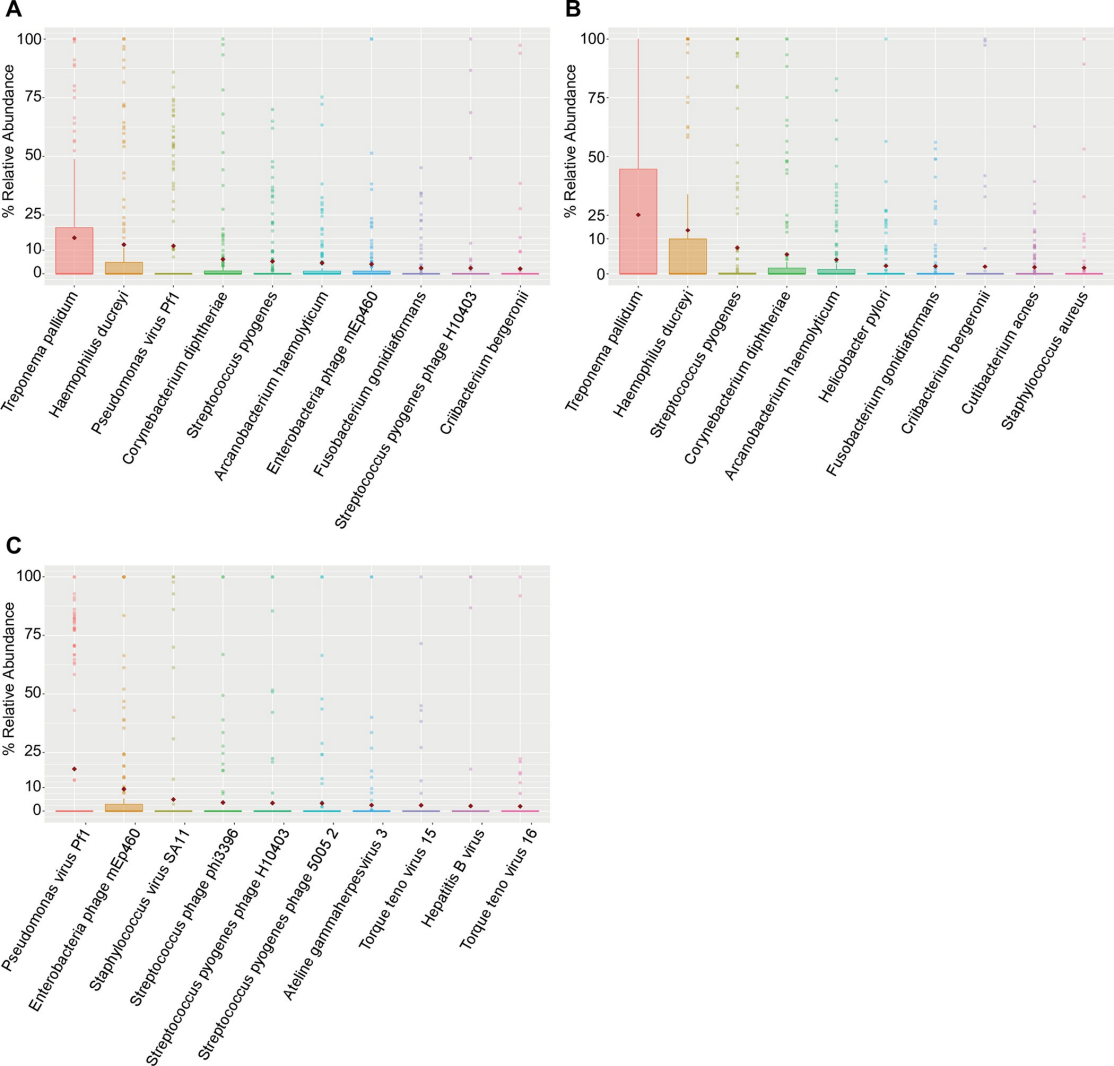
Top 10 most relatively abundant taxa from ulcers in the stringent dataset. Box and whisker plots using (A) all identified taxa, (B) only bacterial taxa, and (C) only viral taxa. All box plots show medians with hinges corresponding to the 25^th^ and 75^th^ percentiles and whiskers extending no further than 1.5x interquartile range from the hinges. Red diamonds signify means. Medians are shifted toward zero due to the absence of many of the taxa in the majority of the specimens (N = 139).

The 244 CU specimens yielded a median depth of 6.5 GB of DNA sequence per specimen. In the stringent dataset, we identified 149 unique microbial species– 83 bacterial, one eukaryotic, and 65 viral species. The relative abundance of these species across all CU specimens were 57.74%, 0.03%, and 42.23%, respectively. In the overall dataset, we identified 192 unique microbial species– 110 bacterial, one eukaryotic, and 81 viral species. The relative abundance of these species across all CU specimens were 50.55%, 0.04%, and 49.41%, respectively. For further analyses, we separated species by kingdom, with a focus on bacterial and viral kingdoms. A summary of the most abundant taxa identified for the stringent and overall datasets are shown in Figs 1 and S1, respectively.

**Fig 2:**
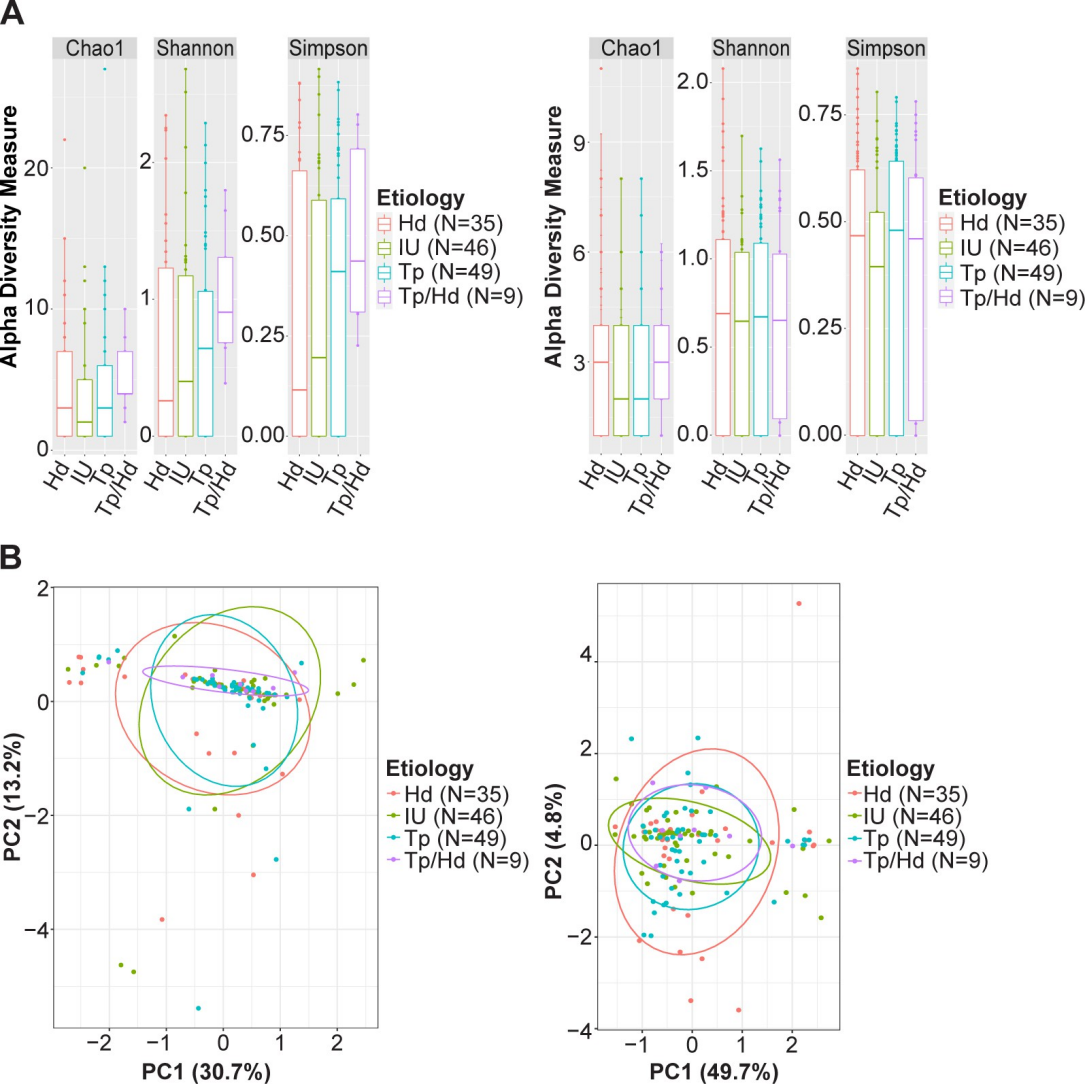
Alpha and beta diversity estimates in the stringent dataset. (A) Box plots showing Chao1 richness estimate and Shannon and Simpson diversity indices for bacterial (left) and viral (right) taxa from ulcers. No significant differences were observed (P > 0.05). (B) Principal component analyses of bacterial (left) and viral (right) taxa from ulcers. Data was first transformed using additive log ratio with human reads as the invariant taxon. All PERMANOVA comparisons of groups using only bacterial taxa were significantly different (P < 0.05), while no comparisons of groups using only viral taxa were significant (P > 0.05).

Microbiome data is inherently compositional, so the taxon count data was transformed with an additive log ratio (ALR) method, using the number of human reads in each specimen as the invariant taxon. Viral and bacterial alpha diversity were similar in both the stringent and overall datasets (Figs 2A and S2A); however, community compositions differed between HD+, TP+, HD+TP+, and IU groups (Figs 2B and S2B) when all microorganisms or all bacteria sequences were compared, but not when only the viral sequences were compared between groups.

Next, we separated the CU samples by timepoint (36, 42, and 48 months) and performed alpha diversity measures. No differences were detected in any alpha diversity measure in the overall or stringent datasets. When we examined changes in diversity over time by ulcer classification (HD+, TP+, TP+/HD+, IU), differences were detected in the IU group in the stringent dataset. From 36 to 48 months, the relative abundance of *S*. *pyogenes* decreased, while the relative abundances of *C*. *bereronii* and *F*. *necrophorum* increased (S4 Table). The increase in *C*. *bereronii* was significant between 36 and 48 months (P = 0.04) and trended towards significance between 42 and 48 months (P = 0.06). These observations suggested that anaerobes were replacing *S*. *pyogenes* during the study, perhaps due to the continued use of azithromycin to treat persons with ulcers as part of the Morges Strategy.

We also used the Calinski-Harabasz (CH) Index to determine the optimal number of clusters into which the CU specimens could be separated based upon the sequencing results. As opposed to our separation of specimens into four groups by PCR-based etiology, for the stringent dataset, CH index analysis determined that two clusters were optimal when only the bacterial sequences or bacterial and viral sequences were considered (S3A Fig). When only bacterial sequences were considered, one cluster contained all the TP+ specimens and two-thirds of the TP+HD+ specimens while the other cluster contained all the HD+ and IU specimens and one-third of the TP+HD+ specimens (S3B Fig). No obvious trends were observed when only the viral reads were considered (S3B Fig) or in the overall dataset (S4 Fig). These data suggest that 1) IU specimens may be closer in composition to HD+ specimens than TP+ specimens and possibly TP+HD+ specimens and 2) the microbiome of TP+ specimens is unique. Given that these two different approaches to analyzing community composition both show that differences in bacterial but not viral composition are important in distinguishing ulcer etiologies, we subsequently focused only on bacterial data by filtering out the viral species.

To identify bacterial taxa associated with IU, we next examined which bacteria were enriched in the IU compared to those found in the HD+, TP+, and TP+HD+ ulcers. Similar to our 16S rRNA gene sequencing data, *S*. *pyogenes* was the most abundant bacteria in IU in the stringent dataset (24.71% in IU versus 3.17%, 5.86%, and 0.25% for HD+, TP+, and TP+HD+, respectively; P < 0.05 for all comparisons to IU) (Table 1) and overall dataset (24.18% in IU versus 13.32%, 4.27%, and 4.38% for HD+, TP+, and TP+HD+, respectively; p < 0.05 for all comparisons to IU) (S5 Table) and was detected in ~39% of IU specimens. *Staphylococcus aureus* was also enriched or trended towards being enriched in IU in the stringent dataset (5.90% in IU versus 1.07% [p = 0.079], 0.94% [p = 0.051], and 0.00% [p = 0.044] for HD+, TP+, and TP+HD+, respectively). In the overall dataset, *S*. *aureus* enrichment was observed only when comparing IU to TP+HD+ (5.77% versus 0.23%, p = 0.039). The only other bacterial species enriched in IU in the stringent dataset was *Criibacterium bergeronii*, a member of the *Peptostreptococcaceae* family, and this was only observed when comparing IU to HD+ (6.96% versus 0.00%; p = 0.033). Together, these observations suggest that *S*. *pyogenes* is enriched in IU compared to HD+, TP+, and TP+HD+ ulcers.

**Table 1:** Differentially Enriched Bacterial Species in IU.

Species	Comparison	Relative Abundance (%)	P value
*Streptococcus pyogenes*	IU vs HD+	24.71 vs 3.17	0.004
	IU vs TP+	24.71 vs 5.86	0.026
	IU vs TP+HD+	24.71 vs 0.25	0.014
*Criibacterium bergeronii*	IU vs HD+	6.96 vs 0.00	0.033
*Staphylococcus aureus*	IU vs TP+HD+	5.90 vs 0.00	0.044

Abbreviations: IU, Idiopathic Ulcer; HD+, *H*. *ducreyi*; TP+, *T*. *palldium* sub. p*ertenue;* TP+HD+, *H*. *ducreyi* and *T*. *pallidum* sub. *pertenue*

### *emm* typing of *S*. *pyogenes* in IU specimens

The M protein, encoded by the *emm* gene, is one of the major virulence factors of *S*. *pyogenes*, and *S*. *pyogenes* isolates can be typed based on sequence variation in the *emm* gene. The current *emm* typing system is based on matching the 180 nucleotides corresponding to the first 50 residues of the mature M protein and the adjacent 10 C terminal residues of the signal sequence of *emm*. We attempted to bioinformatically identify the *emm* types in the IU specimens with *S*. *pyogenes* by matching cataloged *emm* sequences from the Center for Disease Control and Prevention website to the raw reads. No matching *emm* sequences were detected in these specimens, likely due to the overwhelming amount of human gene contamination (~97% of each ulcer specimen mapped to the human genome). We therefore used a reference-based approach by selecting *S*. *pyogenes* reference genomes based on the single ASV of *S*. *pyogenes* detected in our previous 16S rRNA gene study. We performed a BLAST search for this ASV against all *S*. *pyogenes* assemblies in NCBI and retained the sequences that were perfect matches (100% identity with max scores). Sequences with perfect *emm* gene matches (100% identity and query coverage and no mismatches or gaps) were then removed. This yielded seven strains that we subsequently used as references. We merged the raw reads from the 18 IU specimens containing *S*. *pyogenes* and extracted reads using BWA that aligned to the corresponding seven reference *S*. *pyogenes* genomes. Pseudochromosomes were then assembled from the sequences. Sequences of sufficient length that matched *emm*156 and *emm*166 were detected in each of the pseudochromosomes. Thus, at least two *emm* types of *S*. *pyogenes* are associated with IU on Lihir Island.

### IU specimens that lacked *S*. *pyogenes*

**Fig 3:**
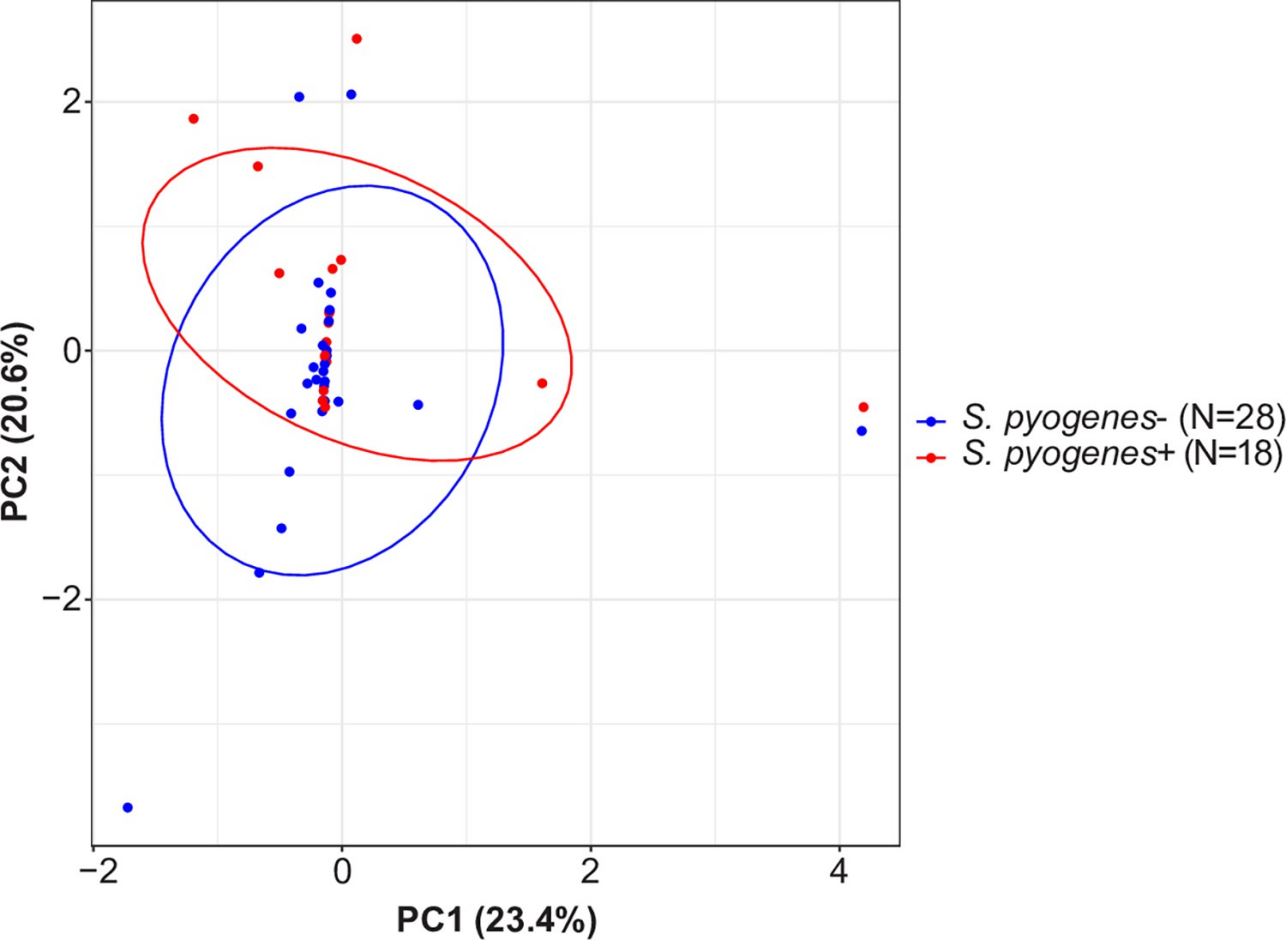
Principal component analysis of idiopathic ulcers grouped by presence or absence of *S*. *pyogenes*. IU specimens in the stringent dataset were sub-grouped based on if they contained *S*. *pyogenes* sequences, followed by transformation using additive log ratio with human reads as the invariant taxon. A PERMANOVA comparison between groups shows a significant difference (P < 0.05).

**Fig 4:**
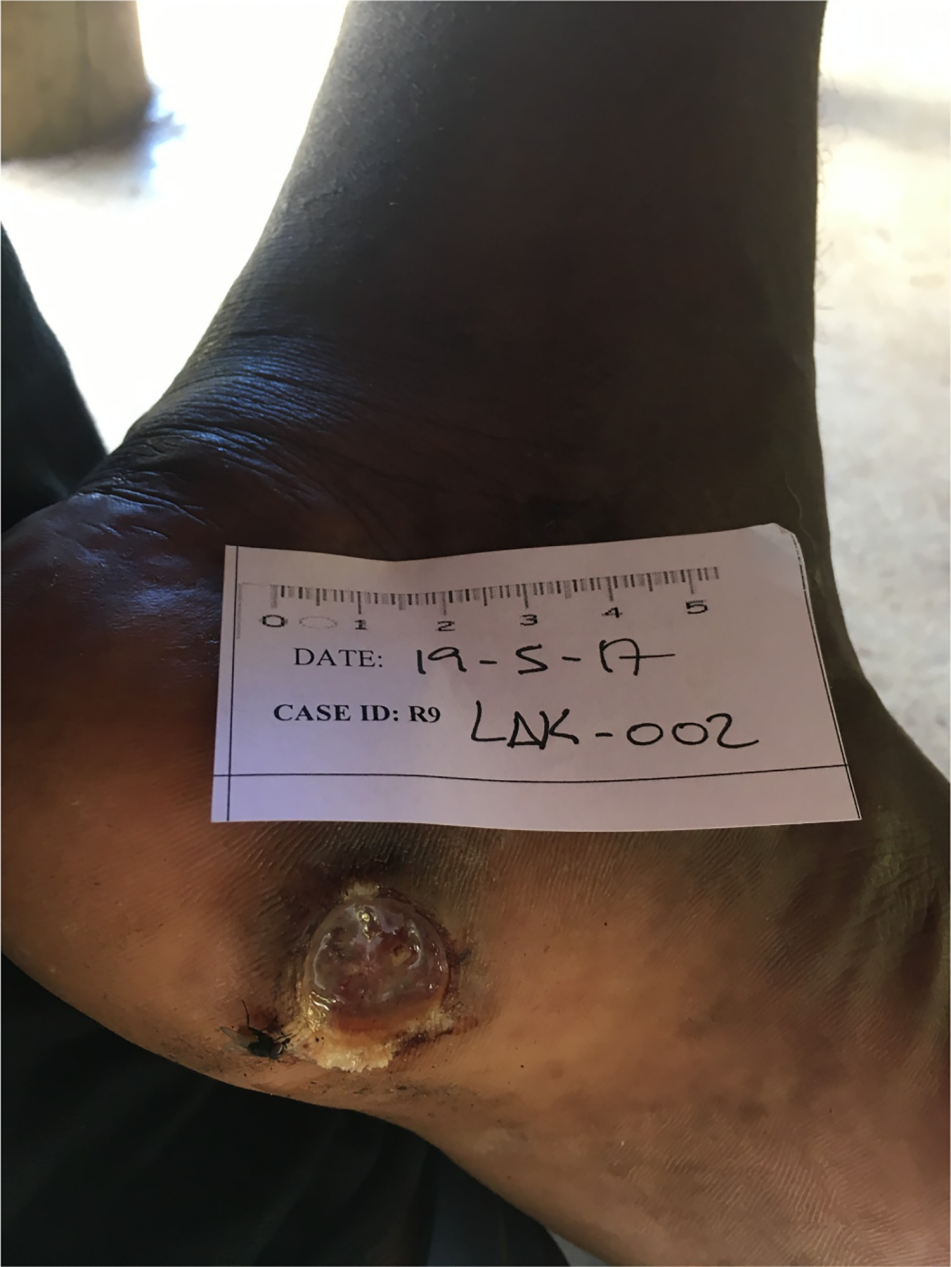
Photograph of a “tropical phagedenic ulcer” from a participant in the yaws eradication campaign. This deep, exudative, and necrotizing ulcer was negative for *T*. *pallidum* sub *pertenue* and *H*. *ducreyi* DNAs by PCR and shotgun sequencing. DNAs for both *F*. *necrophorum* and *Peptostreptococcaceae* were detected by shotgun sequencing.

Only ~39% (18/46) of IU specimens in the stringent dataset contained *S*. *pyogenes*, suggesting that additional microorganisms could be associated with IU. To explore this possibility, we divided the IU group into two subgroups based on the presence or absence of *S*. *pyogenes* reads. PCA plots suggested that the two subgroups are compositionally distinct (p = 0.001) (Figs 3 and S5). Of the species that were detected in the IU subgroup that lacked *S*. *pyogenes*, had a relative abundance greater than 1%, and were found in three or more specimens, *C*. *bergeronii* and *Fusobacterium necrophorum* were significantly enriched compared to the IU subgroup that contained *S*. *pyogenes* (7.07% versus 0.00% [p = 0.001] and 2.18% versus 0.00% [p = 0.014], respectively) (S6 Table). These organisms were found in six and three *S*. *pyogenes* negative IU specimens, respectively. Both *C*. *bergeronii* and *F*. *necrophorum* have been identified previously by culture in what have been called “tropical phagedenic ulcers”, which present as deep, exudative, and necrotizing lesions [33,34]. Interestingly, this clinical presentation occurred in the patients whose ulcers yielded *C*. *bergeronii* and *F*. *necrophorum*. (Fig 4). These observations suggest that, in the absence of *S*. *pyogenes*, other bacteria can colonize and possibly contribute to ulcer formation or maintenance in IU.

## Discussion

Identification of microbes responsible for IU will help with clinical management of exudative cutaneous ulcers originally attributed to yaws. While we have previously shown an association between *S*. *pyogenes* and IU by 16S rRNA sequencing [5], this study precluded identification of non-bacterial organisms and the results could have been skewed by primer bias issues inherent in that technique. Therefore, we performed shotgun metagenomic sequencing on CU specimens that had been previously identified as positive for TP, HD, both, or IU based on PCR testing. We confirmed that *S*. *pyogenes* was highly enriched in IU and that non-bacterial agents are unlikely to be involved in IU pathogenesis.

Similar to 16S rRNA sequencing in our prior study, we observed here that shotgun sequencing was not as sensitive as PCR in detecting HD or TP sequences. Most reads obtained by direct sequencing are from the human host and direct sequencing does not involve amplification of a target, which could explain the lower sensitivity of this approach compared to PCR. We did compare the shotgun sequencing data to our 16S rRNA data. Using the stringent criteria, 116 samples were analyzed in both studies. In each sample, approximately 60% of the bacterial species detected using 16S rRNA sequencing were also found using shotgun sequencing. When we compared bacterial species that had at least a 1% relative abundance in a sample by either sequencing method, the percentage of agreement increases to approximately 80%. In the overall dataset, 237 samples were analyzed in both studies. Approximately 45% of the bacterial species detected in individual specimens using 16S rRNA sequencing were also found in the same specimen using shotgun sequencing. When we compared bacterial species that had at least a 1% relative abundance in at least one of the four ulcer etiologies by either sequencing method, the percentage of agreement increased to approximately 75%. Thus, the data obtained by shotgun sequencing correlated well with but did not exactly match those obtained by 16S rRNA sequencing. These differences are likely explained by subtle differences in what each of these sequencing approach actually measures. 16S rRNA sequencing measures 16S rRNA gene sequences. Copy number of the 16S rRNA alleles can vary between different bacterial species and can vary even in strains of the same bacterial species. 16S rRNA sequencing is also PCR-dependent approach, which can inflate the number of sequence variants due to amplification of random replication errors that occur during the early cycles of amplification. Shotgun sequencing does not involve amplification and randomly breaks up DNA and uses overlapping reads to assemble them into a continuous sequence.

*S*. *pyogenes* is a facultative anaerobe that colonizes the pharynx and the skin and causes diseases such as pharyngitis, impetigo, erysipelas, cellulitis, and necrotizing fasciitis [35]. The only known reservoir for *S*. *pyogenes* is humans; globally, there are ~100 million cases of *S*. *pyogenes* skin infection per year [36,37]. In the South Pacific, where our specimens for this study originated, impetigo is the predominant form of *S*. *pyogenes* infection [38]. Until our studies on the Lihir Island cohort, *S*. *pyogenes* had not been associated with CU.

We hypothesize that either 1) there is/are a clade(s) of *S*. *pyogenes* that have developed the ability to cause CU, 2) *T*. *pallidum* sub *pertenue* and/or *H*. *ducreyi* caused the ulcer, which was subsequently colonized by *S*. *pyogenes* and overgrew *T*. *pallidum* sub *pertenue* and/or *H*. *ducreyi*, or 3) *S*. *pyogenes* emerged as an important cause of CU in a population that had been heavily exposed to azithromycin via the Morges strategy. To test the first hypothesis, we used a bioinformatic approach on a limited number of specimens and found evidence that at least 2 *emm* types are associated with CU on Lihir Island. Due to a lack of infrastructure, cultures are not done on children with pharyngitis or impetigo on Lihir Island; thus, the circulating *emm* types are unknown. Longitudinal analysis of *emm* types in a high prevalence setting of pharyngitis and impetigo in Fiji detected that 19–37 *emm* types circulated during six screening periods conducted every 2 months in school-based settings, that the 5 most frequent *emm* types accounted for only ~30% of the isolates, and that there was no evidence of tissue tropism for the *emm* types [38]. Whether the 2 *emm* types found in our study are unique to CU or reflect the *emm* types circulating on Lihir Island remains to be determined.

Testing of the second hypothesis would be unethical, as this would require a longitudinal study in which treatment would be withheld from children with ulcers. The third hypothesis implies that *S*. *pyogenes* in CU may be macrolide-resistant [39]. *S*. *pyogenes* develops or acquires macrolide resistance primarily through MefA, a drug efflux pump [40], or ribosomal methylation of the 50S ribosomal subunit through ErmB or ErmTR [41] and rarely via 23S rRNA gene mutations or mutations in the ribosomal protein L4 [42]. We attempted to test for macrolide-resistant *S*. *pyogenes* from ulcer specimens bioinformatically, but we could not verify the presence of the *mefA*, *ermB*, and/or the *ermTR* genes; this failure may have been due to use short read technology (2x150 bp) used in this study or the absence of these genes. We did not have sufficient DNA to attempt long read sequencing technologies, which would be better for identifying the possible presence of these genes [43]. Testing of the first and third hypotheses would be facilitated by isolation of *S*. *pyogenes* from children with pharyngitis, impetigo, and CU and performance of whole genome sequencing and antimicrobial susceptibility testing.

Often in acute infections, a single pathogen outgrows the normal flora and causes disease. Our data suggest that *S*. *pyogenes* is associated with IU; however, only 39% of the IU specimens contained detectable *S*. *pyogenes* sequences, suggesting that there are additional causes of IU. Our study identified *C*. *bergeronii* and *F*. *necrophorum* in 9 of 46 (20%) IU cases. *C*. *bergeronii* is a gram-negative, anaerobic bacterium and member of the *Peptostreptococcaceae* family [44]. *F*. *necrophorum* is also a gram-negative, anaerobic bacterium [45]. Both *Peptostreptococcaceae* and *Fusobacterium* have been identified in “tropical phagedenic ulcers” [33,34]. In IU specimens containing either *C*. *bergeronii* or *F*. *necrophorum*, these organisms were highly abundant, suggesting that these bacteria proliferate in the ulcers. Whether these bacteria cause ulcer formation or are secondary infections is unknown. *C*. *bergeronii* and *F*. *necrophorum* do not respond well to azithromycin treatment, which may explain why they survived the antibiotic pressure of the eradication campaign.

Euclidean distance measurements of the ALR-transformed data and our beta diversity measurements revealed that each defined CU etiology is compositionally distinct. Using an unsupervised approach, CH indexing showed that two clusters was optimal, and that TP ulcers clustered separately from HD and IU. Although no histopathological studies have been done on IU, ulcers caused by *T*. *pallidum* sub *pertenue* resemble granulomas based on the predominance of plasma cells [46]. *H*. *ducreyi* ulcers are dominated by neutrophilic infiltrates with accompanying CD4+ and CD8+ T cells [47]. We suspect that the difference in the community compositions of TP and HD ulcers may be driven by the different immune responses to these organisms.

In our previous study using 16S rRNA sequencing, we associated *Campylobacter sputorum* and *Catonella morbi* with IU in specimens that did not contain *S*. *pyogenes* [5]. With shotgun sequencing, we did not detect any *C*. *sputorum*, suggesting a possible misclassification of the sequence occurred in either study. As for *C*. *morbi*, we found this organism only in two IU specimens, both of which did not contain *S*. *pyogenes* (p = 0.111). Thus, the role of *C*. *sputorum* and *C*. *morbi* in CU is unclear.

A previous study used shotgun sequencing on specimens obtained earlier (6 to 18 months post MDA) during the yaws eradication campaign on Lihir Island to identify agents of IU [4]. While *S*. *pyogenes* was detected in CU in that study, its relative abundance was much lower than what we found in this study (<1% for *S*. *pyogenes* in the earlier study vs. 11.05% in this study). The previous shotgun sequencing study identified *Corynebacterium diphtheriae*, *Arcanobacterium haemolyticum*, and *Streptococcus dysgalactiae* as possible bacteria associated with IU. In this study, *C*. *diptheriae* and *A*. *haemolyticum* were the second and sixth most abundant bacterial species in IU (13.79% and 4.53%, respectively), but we did not find any difference between any of these species in IU versus that in other CU groups, suggesting that these species may be colonizing the ulcer but not necessarily causing the ulcer. We did not detect *S*. *dysgalactiae* using shotgun sequencing. The differences observed between the studies may be due to the different specimen collection periods or other technical differences. There was a large difference in sequencing depth between the studies, with this study having approximately 17x more depth than the previous study (11,418 median microorganism reads vs 678 median reads with species level identification in the CU specimens, respectively). Choice of annotation methods between the previous study (MEGAN and Kraken) and our study (MetaPhlAn3) may also explain some differences found in these two studies. Kraken uses K-mers but is limited by errors in underlying databases; many microbial genomes are contaminated with human sequences. MetaPhlAn3 uses exact matches to clade-specific sequences, but uses a much more limited curated database, thus microorganisms can be missed entirely. As well, our study utilized 244 ulcer specimens that were processed specifically to avoid contamination inherent in low abundance microbiome samples while the previous study utilized 122 residual ulcer specimens.

All the possible bacterial agents of IU we found in our study can also be found in the oral cavity and upper respiratory tract. The study was performed using specimens collected on Lihir Island, Papua New Guinea, a rural area that has little access to basic sanitation. Chewing and spitting betel nuts is common on Lihir Island and is a major health concern that has been banned in many parts of Papua New Guinea [48]. Spitting on the ground where children play and the lack of access to basic sanitation and hygiene could promote exposure of the lower legs to oral microorganisms.

One limitation of this study, and all microbiome studies performed on humans, is that we cannot prove causation between a bacterium identified through shotgun sequencing and IU; we can claim only an association. Whether *S*. *pyogenes*, *C*. *bergeronii*, and/or *F*. *necrophorum* are actual causes of ulcers or are a result of multi-microbial succession throughout the development and recovery from a skin ulcer is yet to be determined. A second limitation is that we were only able to identify associations between specific microbes and ~ 59% of cases of IU. Perhaps these pathogen-negative ulcers were caused by organisms that are associated with CU but were in the process of being cleared by the host. Nevertheless, we confirmed that *S*. *pyogenes* is associated with idiopathic ulcers on Lihir Island and found *C*. *bergeronii* and *F*. *necrophorum* to be associated with CU in children who clinically presented with tropical phagedenic ulcers. Importantly, in the stringent dataset, PCR and shotgun sequencing did identify microbes associated with 120 of 139 (86.3%) of cases of CU and provides a basis for modifying PCR-based testing to include *S*. *pyogenes* and anaerobes. Given that there have been no records of penicillin resistance for *T*. *pallidum*, *S*. *pyogenes*, *C*. *bergeronii*, or *F*. *necrophorum*, benzathine penicillin may be the best secondary treatment for CU that fails azithromycin treatment. Future studies need to address the etiology of CU in other populations that have not been exposed to MDA of azithromycin and azithromycin resistance in the bacterial species associated with IU.

## Supporting information

S1 Fig Top 10 most relatively abundant taxa from ulcers in the overall dataset. Box and whisker plots using (A) all identified taxa, (B) only bacterial taxa, and (C) only viral taxa. All box plots show medians with hinges corresponding to the 25^th^ and 75^th^ percentiles and whiskers extending no further than 1.5x interquartile range from the hinges. Red diamonds signify means. Medians are shifted toward zero due to the absence of many of the taxa in the majority of specimens (N = 244).
(TIF)

S2 Fig Alpha and beta diversity estimates from the overall dataset. (A) Box plots showing Chao1 richness estimate and Shannon and Simpson diversity indices for bacterial (left) and viral (right) taxa from ulcers. No significant differences were observed (P > 0.05). (B) Principal component analyses of bacterial (left) and viral (right) taxa from ulcers. Data was first transformed using additive log ratio with human reads as the invariant taxon. All PERMANOVA comparisons of groups using only bacterial taxa were significantly different (P < 0.05), while no comparisons of groups using only viral taxa were significant (P > 0.05).
(TIF)

S3 Fig Unsupervised clustering of bacterial and viral taxa in the stringent dataset. (A) Calinski-Harabasz Index for bacterial (left) and viral (right) taxa from ulcers in the stringent dataset. Two clusters were determined to be the optimal number of clusters for the ulcers using either bacterial or viral taxa. (B) Heatmap showing ulcer clustering results for bacterial (left) and viral (right) taxa from the stringent dataset. Data was first transformed using additive log ratio with human reads as the invariant taxon. Using bacterial taxa, all IU and HD+ ulcers clustered together and separately from TP+ ulcers. No obvious clustering patterns were observed when using viral taxa.
(TIF)

S4 Fig Unsupervised clustering of bacterial and viral taxa in the overall dataset. (A) Calinski-Harabasz Index for bacterial (left) and viral (right) taxa from ulcers from the overall dataset. Three clusters and two clusters were determined to be the optimal number of clusters for the ulcers using either bacterial or viral taxa, respectively. (B) Heatmap showing ulcer clustering results for bacterial (left) and viral (right) taxa from the overall dataset. Data was first transformed using additive log ratio with human reads as the invariant taxon. No obvious clustering patterns were observed when using either bacterial or viral taxa.
(TIF)

S5 Fig Principal component analysis of idiopathic ulcers grouped by presence or absence of *S*. *pyogenes* in the overall dataset. IU specimens in the overall dataset were sub-grouped by presence or absence of *S*. *pyogenes* followed by transformation using additive log ratio with human reads as the invariant taxon. A PERMANOVA comparison between groups shows significant difference (P < 0.05).
(TIF)

S1 Table Specimens and Demographic Information of Children with Ulcers. (DOCX)

S2 Table Shotgun Sequencing Results Grouped by PCR Classification in the Overall Dataset. (DOCX)

S3 Table Shotgun Sequencing Results Grouped by PCR Classification in the Stringent Dataset. (DOCX)

S4 Table Average Relative Abundance (%) of Selected Bacteria in IU Over Time in the Stringent Dataset. (DOCX)

S5 Table Differentially Enriched Bacterial Species in IU in the Overall Dataset. (DOCX)

S6 Table Differentially Enriched Bacterial Species in IU lacking *S*. *pyogenes* in the Stringent Dataset. (DOCX)

S1 Data Relative Abundances of Microbial Taxa Identified in the Stringent Dataset. (XLSX)

S2 Data Relative Abundances of Microbial Taxa Identified in the Overall Dataset. (XLSX)
