# Supplementary figures and images for: Two Streptococcus pyogenes emm types and several anaerobic bacterial species are associated with idiopathic cutaneous ulcers in children after community-based mass treatment with azithromycin
Source: PLoS Negl Trop Dis. 2022 Dec 19;16(12):e0011009. doi: 10.1371/journal.pntd.0011009 (PMC9810193; doi:10.1371/journal.pntd.0011009)

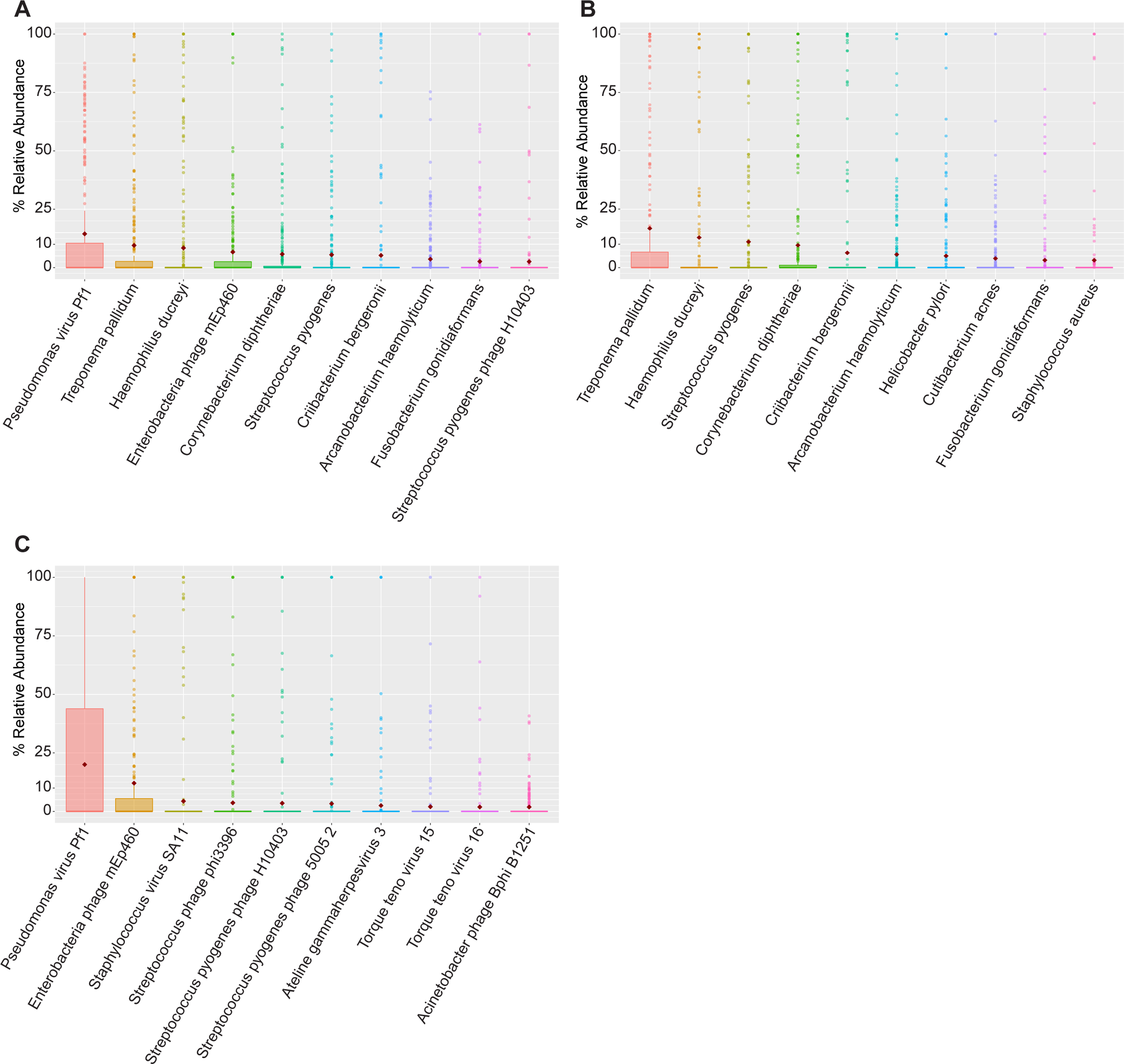

Supplement: S1 Fig — Box and whisker plots using (A) all identified taxa, (B) only bacterial taxa, and (C) only viral taxa. All box plots show medians with hinges corresponding to the 25th and 75th percentiles and whiskers extending no further than 1.5x interquartile range from the hinges. Red diamonds signify means. Medians are shifted toward zero due to the absence of many of the taxa in the majority of specimens (N = 244). (TIF) [file pntd.0011009.s001.tif]

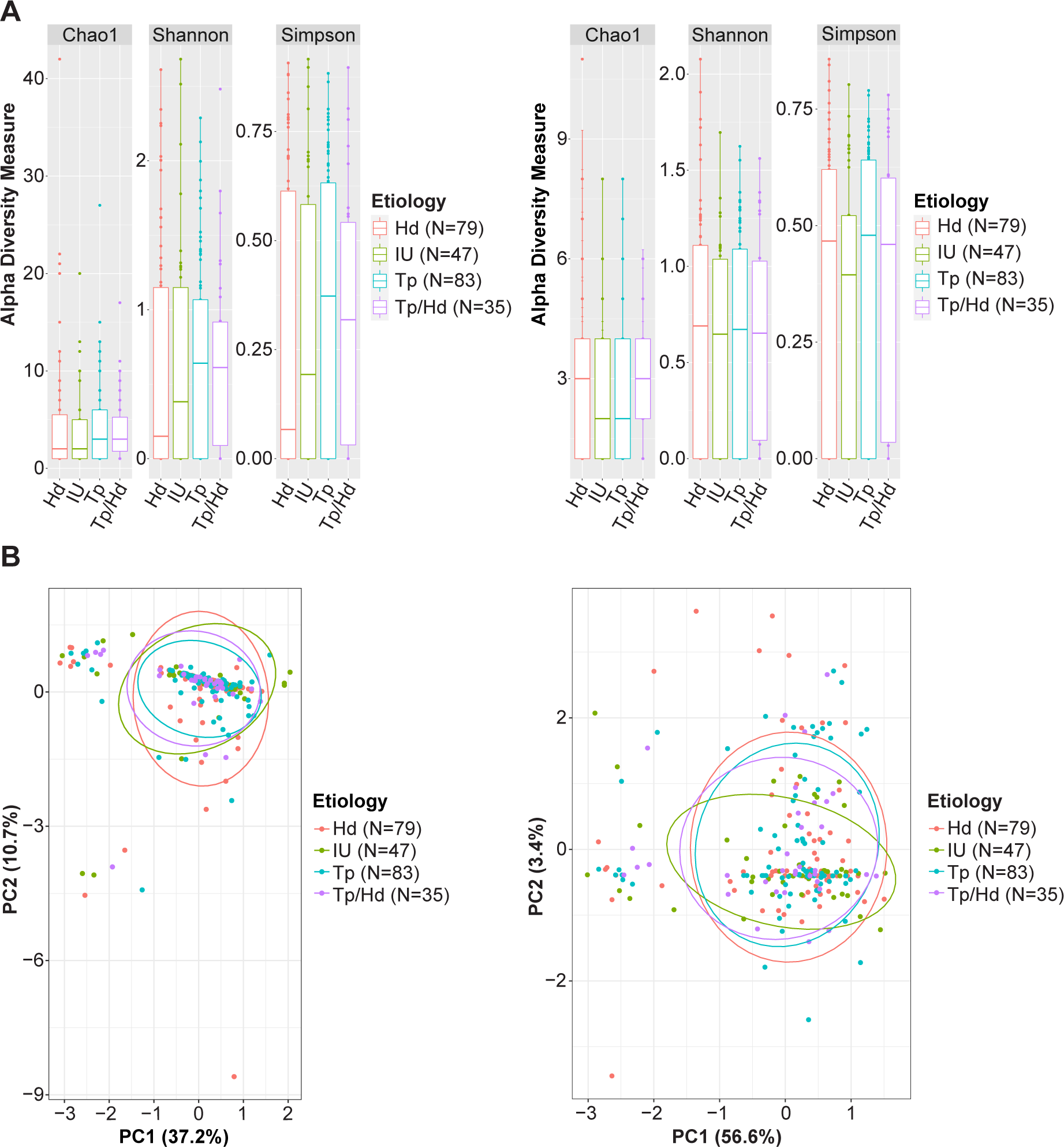

Supplement: S2 Fig — (A) Box plots showing Chao1 richness estimate and Shannon and Simpson diversity indices for bacterial (left) and viral (right) taxa from ulcers. No significant differences were observed (P > 0.05). (B) Principal component analyses of bacterial (left) and viral (right) taxa from ulcers. Data was first transformed using additive log ratio with human reads as the invariant taxon. All PERMANOVA comparisons of groups using only bacterial taxa were significantly different (P < 0.05), while no comparisons of groups using only viral taxa were significant (P > 0.05). (TIF) [file pntd.0011009.s002.tif]

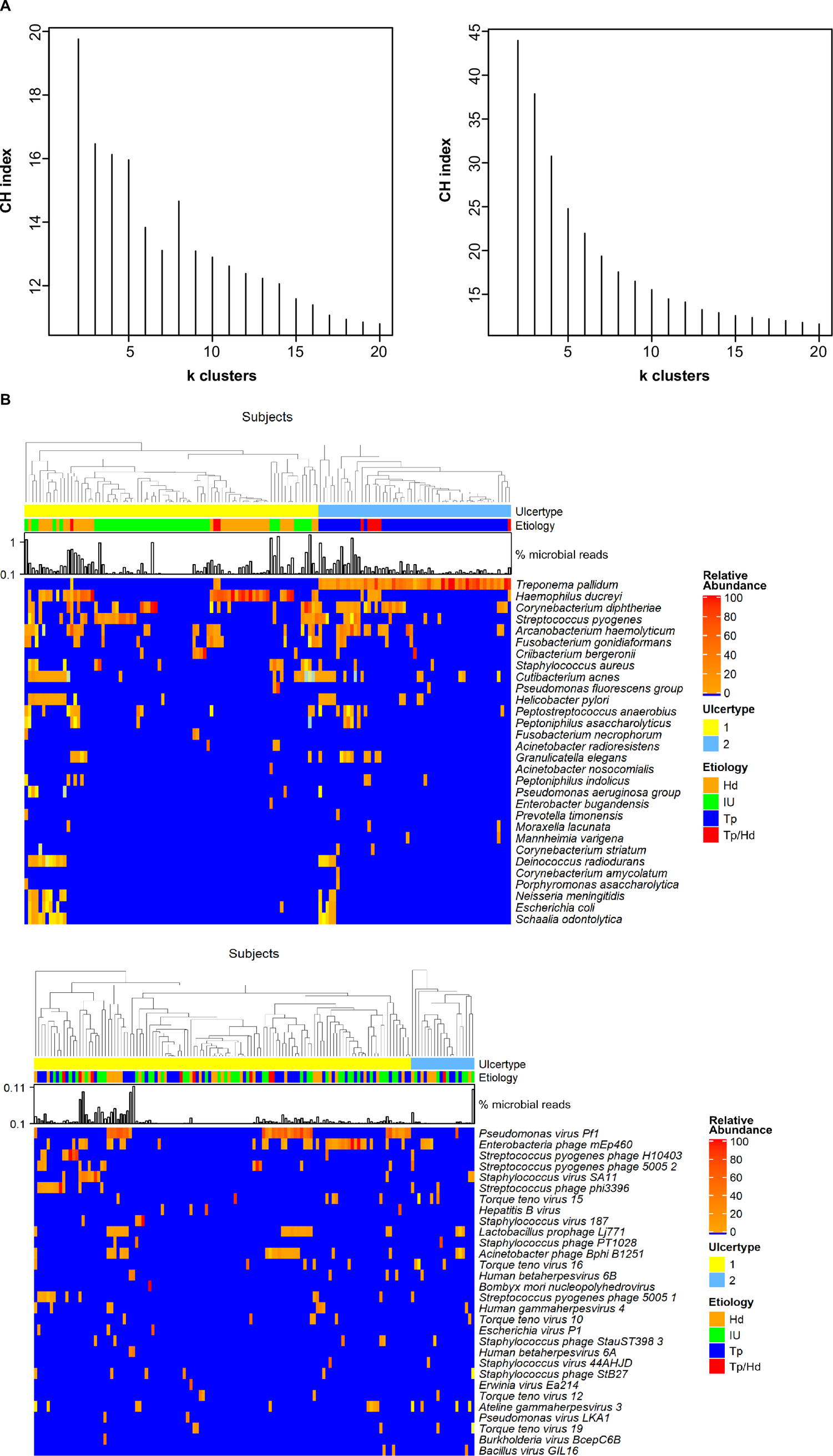

Supplement: S3 Fig — (A) Calinski-Harabasz Index for bacterial (left) and viral (right) taxa from ulcers in the stringent dataset. Two clusters were determined to be the optimal number of clusters for the ulcers using either bacterial or viral taxa. (B) Heatmap showing ulcer clustering results for bacterial (left) and viral (right) taxa from the stringent dataset. Data was first transformed using additive log ratio with human reads as the invariant taxon. Using bacterial taxa, all IU and HD+ ulcers clustered together and separately from TP+ ulcers. No obvious clustering patterns were observed when using viral taxa. (TIF) [file pntd.0011009.s003.tif]

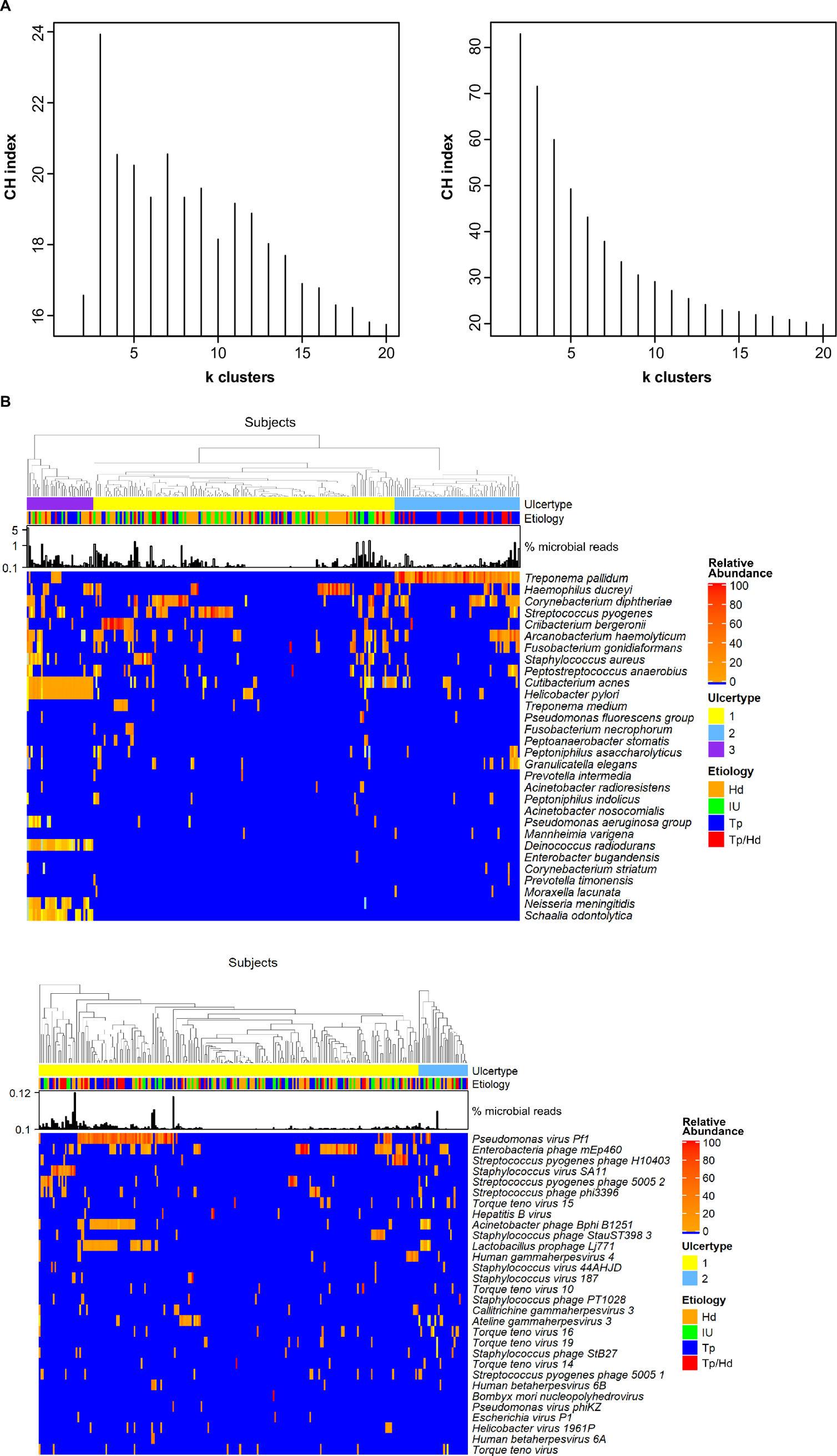

Supplement: S4 Fig — (A) Calinski-Harabasz Index for bacterial (left) and viral (right) taxa from ulcers from the overall dataset. Three clusters and two clusters were determined to be the optimal number of clusters for the ulcers using either bacterial or viral taxa, respectively. (B) Heatmap showing ulcer clustering results for bacterial (left) and viral (right) taxa from the overall dataset. Data was first transformed using additive log ratio with human reads as the invariant taxon. No obvious clustering patterns were observed when using either bacterial or viral taxa. (TIF) [file pntd.0011009.s004.tif]

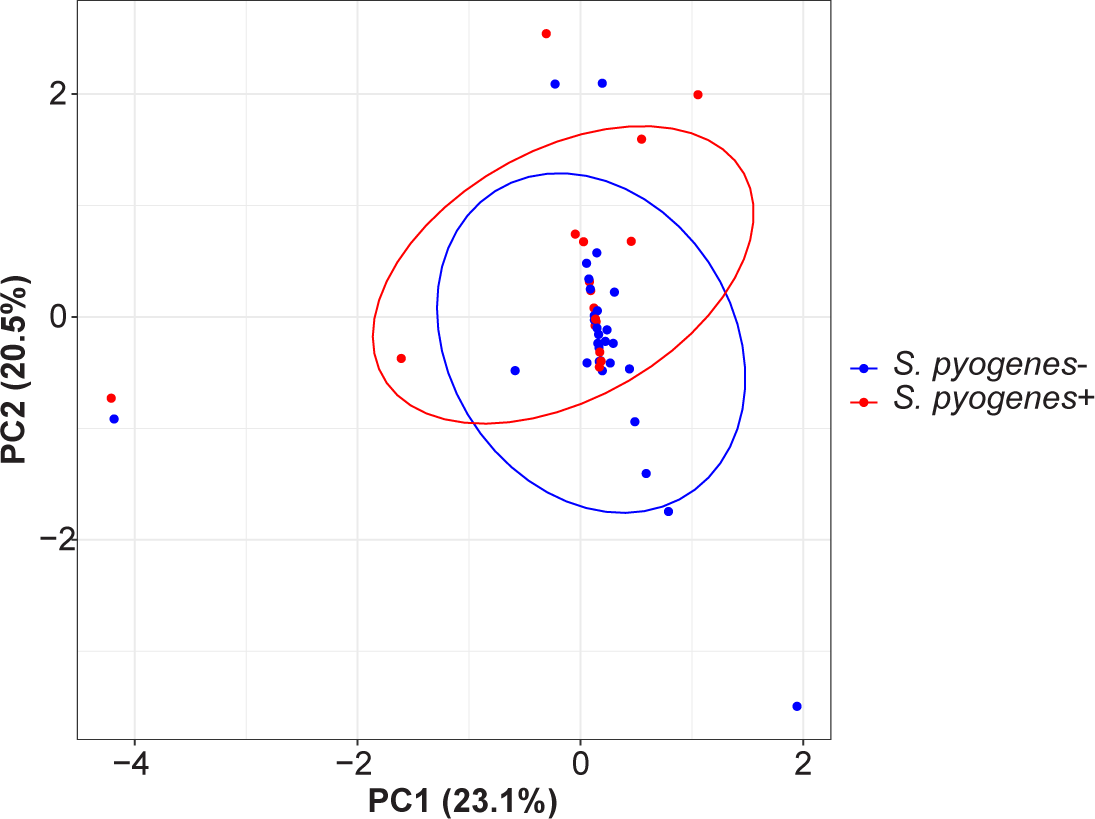

Supplement: S5 Fig — IU specimens in the overall dataset were sub-grouped by presence or absence of S. pyogenes followed by transformation using additive log ratio with human reads as the invariant taxon. A PERMANOVA comparison between groups shows significant difference (P < 0.05). (TIF) [file pntd.0011009.s005.tif]
