# Supplementary material for: Two Streptococcus pyogenes emm types and several anaerobic bacterial species are associated with idiopathic cutaneous ulcers in children after community-based mass treatment with azithromycin
Source: PLoS Negl Trop Dis. 2022 Dec 19;16(12):e0011009. doi: 10.1371/journal.pntd.0011009 (PMC9810193; doi:10.1371/journal.pntd.0011009)
Supplement: S1 Table — (DOCX) [file pntd.0011009.s006.docx]

S1 Table. Specimens and Demographic Information of Children with Ulcers

| **Number of Specimens** | | | | |
| --- | --- | --- | --- | --- |
| **Time post MDA** | **36**  **mo.** | **42**  **mo.** | **48**  **mo.** | **Total (%)** |
|  | 106 | 107 | 66 | 279 |
| **Demographics^a^** | | | | |
| Male | 62 | 64 | 38 | 164 (59) |
| Female | 44 | 43 | 26 | 113 (41) |
| 1-5 yrs. | 15 | 18 | 7 | 40 (14) |
| 6-11 yrs. | 56 | 69 | 37 | 162 (59) |
| 12-17 yrs. | 26 | 14 | 15 | 55 (20) |
| 18+ yrs. | 9 | 6 | 5 | 20 (7) |

^a^, demographic information missing on 2 participants

This table was modified from reference (1)

**Reference**

1. Griesenauer B, Gonzalez-Beiras C, Fortney KR, Lin H, Gao X, Godornes C, et al. *Streptococcus pyogenes* Is Associated with Idiopathic Cutaneous Ulcers in Children on a Yaws-Endemic Island. mBio. 2021;12(1):e03162-20.
