# Supplementary material for: Two Streptococcus pyogenes emm types and several anaerobic bacterial species are associated with idiopathic cutaneous ulcers in children after community-based mass treatment with azithromycin
Source: PLoS Negl Trop Dis. 2022 Dec 19;16(12):e0011009. doi: 10.1371/journal.pntd.0011009 (PMC9810193; doi:10.1371/journal.pntd.0011009)
Supplement: S2 Table — (DOCX) [file pntd.0011009.s007.docx]

| **S2 Table. Shotgun Sequencing Results Grouped by PCR Classification in the Overall Dataset** | | | | | | | | |
| --- | --- | --- | --- | --- | --- | --- | --- | --- |
| **PCR Classification** | **HD+** | | **TP+** | | **TP+/HD+** | | **TP-/HD- (IU)** | |
| **HD or TP Reads** | **HD** | **TP** | **HD** | **TP** | **HD** | **TP** | **HD** | **TP** |
| **Mean Read Count** | 10,104 | 0 | 24 | 4050 | 4959 | 3293 | 10 | 0 |
| **Mean Relative Abundance** | 19.7% | 0.0% | 0.9% | 22.4% | 11.6% | 13.1% | 0.3% | 0.0% |
| **Number of samples positive by sequencing \| Number of samples as classified by PCR** | 35\|79 | 0\|79 | 1\|83 | 51\|83 | 13\|35 | 17\|35 | 1\|47 | 0\|47 |
| Abbreviations: PCR – polymerase chain reaction; HD – *H. ducreyi*; TP – *T. pallidum* sub. *pertenue*;  TP/HD – *T. pallidum* sub. *pertenue* and *H. ducreyi*; IU – Idiopathic Ulcer | | | | | | | | |
