# Supplementary material for: Two Streptococcus pyogenes emm types and several anaerobic bacterial species are associated with idiopathic cutaneous ulcers in children after community-based mass treatment with azithromycin
Source: PLoS Negl Trop Dis. 2022 Dec 19;16(12):e0011009. doi: 10.1371/journal.pntd.0011009 (PMC9810193; doi:10.1371/journal.pntd.0011009)
Supplement: S3 Table — (DOCX) [file pntd.0011009.s008.docx]

| **S3 Table. Shotgun Sequencing Results Grouped by PCR Classification in the Stringent Dataset** | | | | | | | | |
| --- | --- | --- | --- | --- | --- | --- | --- | --- |
| **PCR Classification** | **HD+** | | **TP+** | | **TP+/HD+** | | **TP-/HD- (IU)** | |
| **HD or TP Reads** | **HD** | **TP** | **HD** | **TP** | **HD** | **TP** | **HD** | **TP** |
| **Mean Read Count** | 22,805 | 0 | 0 | 6856 | 4664 | 9794 | 0 | 0 |
| **Mean Relative Abundance** | 44.4% | 0.0% | 0.0% | 37.9% | 18.7% | 30.2% | 0.0% | 0.0% |
| **# of samples positive by shotgun sequencing / # of samples as classified by PCR** | 35/35 | 0/35 | 0/49 | 49/49 | 9/9 | 9/9 | 0/46 | 0/46 |
| Abbreviations: PCR – polymerase chain reaction; HD – *H. ducreyi*; TP – *T. pallidum* sub. *pertenue*;  TP/HD – *T. pallidum* sub. *pertenue* and *H. ducreyi*; IU – Idiopathic ulcer | | | | | | | | |
