# Supplementary material for: Two Streptococcus pyogenes emm types and several anaerobic bacterial species are associated with idiopathic cutaneous ulcers in children after community-based mass treatment with azithromycin
Source: PLoS Negl Trop Dis. 2022 Dec 19;16(12):e0011009. doi: 10.1371/journal.pntd.0011009 (PMC9810193; doi:10.1371/journal.pntd.0011009)
Supplement: S4 Table — (DOCX) [file pntd.0011009.s009.docx]

| **S4 Table. Average Relative Abundance (%) of Selected Bacteria in IU Over Time in the Stringent Dataset** | | | |
| --- | --- | --- | --- |
| **Bacterial Species** | **Months Post MDA (# of ulcers)** | | |
|  | **36 (n=16)** | **42 (n=16)** | **48 (n=14)** |
| *S. pyogenes* | 36.72% | 24.55% | 11.15% |
| *C. bergeronii* | 0.67% | 2.05% | 19.74% |
| *F. necrophorum* | 0.00% | 2.18% | 3.13% |
| Abbreviations: MDA – mass drug administration; IU – idiopathic ulcer | | | |
