# Supplementary material for: Two Streptococcus pyogenes emm types and several anaerobic bacterial species are associated with idiopathic cutaneous ulcers in children after community-based mass treatment with azithromycin
Source: PLoS Negl Trop Dis. 2022 Dec 19;16(12):e0011009. doi: 10.1371/journal.pntd.0011009 (PMC9810193; doi:10.1371/journal.pntd.0011009)
Supplement: S5 Table — (DOCX) [file pntd.0011009.s010.docx]

| **S5 Table. Differentially Enriched Bacterial Species in IU in the Overall Dataset** | | | |
| --- | --- | --- | --- |
| **Species** | **Comparison** | **Relative Abundance (%)** | **P value** |
| *Streptococcus pyogenes* | IU vs HD+ | 24.18 vs 13.32 | 0.043 |
|  | IU vs TP+ | 24.18 vs 4.27 | 0.007 |
|  | IU vs TP+HD+ | 24.18 vs 4.38 | 0.002 |
|  |  |  |  |
| *Staphylococcus aureus* | IU vs TP+HD+ | 5.77 vs 0.23 | 0.039 |
| IU: Idiopathic Ulcer, HD+: *H. ducreyi*, TP+: *T. palldium* sub. *pertenue*, TP+HD+: *H. ducreyi* and *T. pallidum* sub. *pertenue* | | | |
