# Supplementary material for: Two Streptococcus pyogenes emm types and several anaerobic bacterial species are associated with idiopathic cutaneous ulcers in children after community-based mass treatment with azithromycin
Source: PLoS Negl Trop Dis. 2022 Dec 19;16(12):e0011009. doi: 10.1371/journal.pntd.0011009 (PMC9810193; doi:10.1371/journal.pntd.0011009)
Supplement: S6 Table — (DOCX) [file pntd.0011009.s011.docx]

| **S6 Table. Differentially Enriched Bacterial Species in IU lacking *S. pyogenes* in the Stringent Dataset** | | |
| --- | --- | --- |
| **Species** | **Relative Abundance (%)** | **P value** |
| *Criibacterium bergeronii* | 7.07 vs 0.00 | 0.001 |
|  |  |  |
| *Fusobacterium necrophorum* | 2.18 vs 0.00 | 0.014 |
